# A Study on Physical Exercise and General Mobility in People with Cerebral Palsy: Health through Costless Routines

**DOI:** 10.3390/ijerph18179179

**Published:** 2021-08-31

**Authors:** Alberto J. Molina-Cantero, Manuel Merino-Monge, Juan A. Castro-García, Thais Pousada-García, David Valenzuela-Muñoz, Juan Gutiérrez-Párraga, Setefilla López-Álvarez, Isabel M. Gómez-González

**Affiliations:** 1 Departamento de Tecnología Electrónica, E.T.S.I. Informática, Campus de Reina Mercedes, Universidad de Sevilla, 41012 Sevilla, Spain; almolina@us.es (A.J.M.-C.); manmermon@dte.us.es (M.M.-M.); 2 TALIONIS Research Group, CITIC Research Center, University of A Coruña, 15006 Coruña, Spain; thais.pousada.garcia@udc.es; 3 Asociación Sevillana de Parálisis Cerebral (ASPACE), Dos Hermanas, 41704 Seville, Spain; aspacesevilla@aspacesevilla.org; 4 Centro Específico de Educación Especial Mercedes Sanromá, Junta de Andalucía, Camino del Silo, 2, 41012 Seville, Spain; juan.gutierrez@juntadeandalucia.es (J.G.-P.); setefilla03@gmail.com (S.L.-Á.)

**Keywords:** cerebral palsy, physical activity, exercise programs, rehabilitation, low-cost solutions

## Abstract

Sedentary behavior (SB) is a common problem that may produce health issues in people with cerebral palsy (CP). When added to a progressive reduction in motor functions over time, SB can lead to higher percentages of body fat, muscle stiffness and associated health issues in this population. Regular physical activity (RPA) may prevent the loss of motor skills and reduce health risks. In this work, we analyzed data collected from 40 people (20 children and teenagers, and 20 adults) who attend two specialist centers in Seville to obtain an up-to-date picture regarding the practice of RPA in people with CP. Roughly 60% of the participants showed mostly mid/severe mobility difficulties, while 38% also had communicative issues. Most of the participants performed light-intensity physical activity (PA) at least once or twice a week and, in the majority of cases, had a neutral or positive attitude to exercising. In the Asociación Sevillana de Parálisis Cerebral (ASPACE) sample test, the higher the International Classification of Functioning, Disability and Health (ICF), the higher the percentage of negative responses to doing exercise. Conversely, in the Centro Específico de Educación Especial Mercedes Sanromá (CEEEMS), people likes PA but slightly higher ratios of positive responses were found at Gross Motor Function Classification System (GMFCS) levels V and II, agreeing with the higher personal engagement of people at those levels. We have also performed a literature review regarding RPA in CP and the use of low-cost equipment. As a conclusion, we found that RPA produces enormous benefits for health and motor functions, whatever its intensity and duration. Costless activities such as walking, running or playing sports; exercises requiring low-cost equipment such as elastic bands, certain smartwatches or video-games; or therapies with animals, among many others, have all demonstrated their suitability for such a purpose.

## 1. Introduction

Technological solutions are often required to help people with disabilities to become less dependent and improve their quality of life (QoL), which does not just involve aspects such as communication through human-machine interaction (HMI); it also covers other issues related, for example, to health and wellness, where regular physical activity (RPA), as part of a rehabilitative program or a daily routine, is a noteworthy example. According to the World Health Organization (WHO), when people conduct RPA, the risk of suffering from cardiopathies, diabetes, high blood pressure, breast and colon cancers diminishes [1]. WHO guidelines on exercise for young people and adults state that an adult without disabilities needs 150 min of moderate to vigorous aerobic exercise each week [2].

In the past, researchers thought that physical activity (PA) was not appropriate for this group, which was founded on the belief that PA could increase spasticity and muscular stiffness, leading instead to an overall reduction in mobility. However, several studies have found no evidence of such a change in spasticity levels [3]. Indeed, an improvement was observed in spasticity when a rehabilitation exercise was targeted to a specific muscular group [4]. Moreover, a systematic review demonstrated that strengthening interventions produce significant improvements in strength and physical performance for individuals with CP [5].

In a longitudinal study [6], which was carried out over several years with children with CP, researchers observed a reduction in motor abilities during development. This worsening was notable in those with the highest motor limitations, who were initially classified at levels III, IV, and V, according to the GMFCS [7]. Based on the previous evidence, authors suggested RPA to prevent the loss of motor skills while growing.

People with CP present high rates of epilepsy [8], hypertension, diabetes, strokes [9] and, consequently, have higher mortality ratios than the general population [10]. RPA increases cardiorespiratory capacity and may mitigate the effect that other diseases that affect the circulatory and digestive systems produce in this group [9].

In order to treat the movement and posture abnormalities in children with CP, different rehabilitative approaches have been applied, including neurodevelopmental techniques such as hydrotherapy, gross motor task training, hippotherapy, treadmill training (with/without body weight support) and virtual reality, among others [11]. In specialized centers, people with CP can receive physiotherapy to improve their independence and social participation. In most cases, the time scheduled in such centers does not reach the WHO recommendations for exercise [12]. Thus, it is important to provide these people with appropriate, affordable technological solutions so that they can continue conducting RPA according to their physiotherapist’s guidelines in a more autonomous way, but still adapted to their motor capabilities.

In this paper, we attempted to gauge the current situation regarding RPA in people with CP in two specialist CP centers in Seville that care for children and adults with CP in a wide set of activities that include rehabilitation, education and occupational therapy, among others. We wanted to quantify participants’ RPA levels in relation to their motor capabilities, identify the reasons for a lack of PA, present the results globally and propose techniques, tools and low-cost solutions to deal with this problem, promote PA when possible and ensure that participants adhere to a specific program through long-term follow-up.

This manuscript contains three different studies. Study 1 shows a picture of people with CP according to their capabilities and in the context of two specialized centers in Seville. Study 2 is focused on determining the PA conducted by participants in Study 1, comprising the frequency, level and type of activity and motivation, among others. Study 3 shows the results of a scoping review that gathered activities and physical exercises that are recommended to improve muscular strength and fitness.

The paper is organized as follows: Section 2 and Section 3 present the three aforementioned studies. These studies are presented following the typical structure of a scientific communication and were conducted during the last quarter of 2020 and the beginning of 2021. Finally, Section 5 and Section 6 present a global discussion and the conclusions, respectively. This research was approved by the Ethics Committee A Coruña-Ferrol with ID 2020/597 and the boards of the participating centers after they had been duly informed of its objectives.

## 2. Study 1: Global Perspective of the Population

Any project aimed at improving the QoL of a group of people should start by studying their daily routines to detect their needs and propose solutions that are both appropriate and realistic. Such a population description needs to be systematized by using the appropriate scales. Usually, three inter-related areas must be included in a population description [13]:

1. Deficiencies in body structures and functions—this is the point which is assessed more methodologically;
2. Activities performed by the person and their limitations—this aspect is less technical and based on observation;
3. Interaction with the environment—this refers to the supporting context needed for their interaction: caregivers, technical aids, etc.

According to [14], the assessment process must include, among others, the following features: (1) a distinction between the need for assistive technology (AT) and human support; (2) the selection of the context in which the activities under assessment are going to be performed; (3) the scale of the degree of severity; (4) the facilitation of the statistical comparison of results; and (5) a useful tool for programming individual or group interventions, leading to more autonomy.

### 2.1. Participants

The population who took part in this study attend two different centers for people with special needs: Asociación Sevillana de Parálisis Cerebral (ASPACE) and Centro Específico de Educación Especial Mercedes Sanromá (CEEEMS).

ASPACE is a private organization catering mainly for adults with CP. They use the ICF scale for a complete description of their members. The other center, CEEEMS, is a public specialist school that forms part of the educational network in Andalusia (Spain) and deals mainly with children and teenagers with motor dysfunctions (including CP). Unlike ASPACE, they use the GMFCS, Communication Function Classification System (CFCS) and Manual Ability Classification System (MACS) to describe their students.

We collected a sample of 40 participants: 20 from CEEEMS and 20 adults from ASPACE. They all (or their legal representatives) agreed to take part in this study. The sample represented all the profiles of users with CP who attended the centers, with a resulting percentage of around 20% of their members. According to Cochran’s formula [15], this sample size, with p = q = 0.5 (maximal variance) and a confidence interval of 95%, has a margin of error of 13.9%. Lower error margins require a greater sample size. Namely, for a margin of error of 5%, it would have been necessary to recruit more than 130 respondents. However, due to the overworking of the staff in those centers during the pandemic, the implementation of higher sample sizes was not possible. The study was conducted during the last quarter of 2020 and the beginning of 2021.

Participants had to fulfill the following inclusion criteria: (1) they suffered from CP, (2) they did not have other diseases that prevented them from the practice of RPA and (3) the sample had to be representative of the population who attends the centers.

### 2.2. Methods

This section contains the descriptions of the participants who took part in the study and fulfilled the inclusion criteria, a brief description of the centers that care for them, the scales used to assess their motor and communication capabilities and an analysis to find their relationships.

#### 2.2.1. Instruments and Variables: The ICF Scale at ASPACE

The International Classification of Functioning, Disability and Health (ICF) [16] is a “universal framework for the definition, measurement and policy formulations for health and disability”, developed by the WHO and used in health-related sectors.

The ICF is divided into two parts that assess the three areas explained above at the beginning of Section 2:

1. Functioning and disability
  (a) Body functions and structures
  (b) Activities and participation
2. Contextual factors
  (a) Environmental factors
  (b) Personal factors

ASPACE had previously analyzed their members and applied the ICF scale, selecting those items that were considered useful for the development of their daily activities and that encompassed different aspects of users’ lives. For the scope of this work, we used only those items related to their motor functionalities and communicative skills. Table 1 contains a complete description of the relevant items extracted from the ICF used to obtain the ASPACE information.

#### 2.2.2. Instruments and Variables: GMFCS, MACS and CFCS Scales at CEEEMS

The GMFCS [7] is a five-level scale focused on truncal control and walking. The discrimination at each level of motor function is based on functional limitation and the use (or not) of assistive devices such as walkers, wheelchairs, etc. GMFCS could be covered by the ICF, specifically in items chapter 7/b7, related to neuromusculoskeletal and movement functions, and chapter 4/d4, dedicated to mobility.

The five levels into which this scale is divided classify the mobility capacity of people in a way that is significant for their daily life. These levels are described as follows:

Level I Ability to ambulate;
Level II Independently ambulates with limitations;
Level III Ambulates with walking aids;
Level IV Independently mobile with powered mobility;
Level V Dependent on AT for all mobility.

The MACS scale [17] assesses how children use both hands in situations of their daily life and whether they are independent or need some support. The opinion of people who know them and the age of the children are taken into account for this scale. The ICF mobility task chapter 4/d4 mainly covers hand use aspects.

The five-level MACS scale is described as follows:

Level I Handles objects easily and successfully;
Level II Handles most items. Slight reduction in achievement quality and/or speed;
Level III Handles objects with difficulty and needs help;
Level IV Manipulates a limited selection of objects in adapted situations;
Level V Does not manipulate objects. Limited ability to perform simple actions.

The CFCS scale [18] is a classification system for functional communication divided into five levels to identify performance in everyday communication. The CFCS could be covered by chapter 3/b3—voice and speech functions (within personal factors—and chapter 3/d3—communication (within the factors of activities and participation)—of the ICF. The communication process includes not only speech but also gestures, looks and behaviors, as well as the use of augmentative and alternative communication systems. Different actors are involved in the communication process—the sender and the receiver—together with the variable of whether the interlocutor is known or unknown to the evaluated person. The levels are described as follows:

Level I Efficient sender and receiver with known and unknown interlocutors;
Level II Efficient sender and/or receiver, but with a slower pace with known and/or unknown interlocutors;
Level III Efficient sender and efficient receiver with known interlocutors;
Level IV Inconsistent sender and/or receiver with known interlocutors;
Level V Sender and receiver rarely effective even with known interlocutors.

#### 2.2.3. Homogenization of Scales

In this work, we included the same scales that the centers use to describe their members. As explained above, such scales are globally accepted for free use and, in particular, the ICF is backed by the WHO. We mapped the assessment in the ICF performed in ASPACE into the items described in the scales used in CEEEMS to make the assessments more homogeneous between the young and adult population.

#### 2.2.4. Data Analysis

Non-parametric tests were applied in order to analyze the possible relation between variables: Spearman correlation for the quantitative variables, the U Mann–Whitney or Kruskal–Wallis tests for determining the differences in means, and the chi-square for the qualitative variables. All hypothesis tests were set at p<0.05.

### 2.3. Results

Table 2 shows the results of the assessment in the CEEEMS with the scales GMFCS, CFCS and MACS. With respect to GMFCS, 40% of the cases presented the most severe mobility difficulty (level V), 15% were in level IV, 20% of children were in the middle term (level III), and the remaining 25% were in level II. Most people in this sample set had mobility problems. In particular, 75% needed wheelchairs, walking aids or other mechanical equipment to ambulate.

Although mobility was the key point in this study, other aspects related to communication and fine motor skills were no less important as they could be determinant when selecting the most suitable type of PA. For example, if a collaborative game was considered as a motivation for PA, players had to have sufficient communicative capabilities for the game to be considered appropriate for them. We found that 35% of users in the sample set had grades I and II, 40% had grade III and the remaining 25% represented the most severe cases. In short, 75% of the participants analyzed had sufficient communicative capabilities.

**Figure 1:**
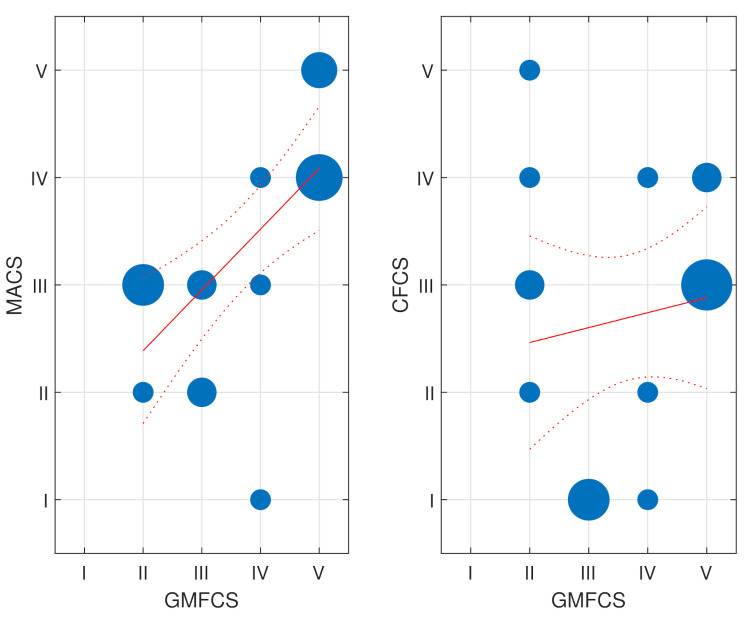
On the (**left**), a bubble chart comparing the GMFCS (gross motor) to MACS (fine motor) for the assessed population in CEEEMS. On the (**right**), the bubble chart comparing the GMFCS (gross motor) to CFCS (communication). The sizes of the blue circles are proportional to the number of occurrences. The linear regression (solid line) and the confidence intervals (dotted line) are also shown in red.

Figure 1 shows the influence of GMFCS on the MACS and CFCS scales. On the left, the MACS and GMFCS scales are compared, showing that as the GMFCS increases, so does the MACS. The relationship was significant according to the linear regression test (*p*-value = 0.002). This result was expected: people with severe gross motor dysfunctions also have difficulty with fine movements. More interestingly, on the other hand, was the absence of a relationship between the CFCS and GMFCS (*p*-value = 0.54), meaning that the communication capabilities did not seem to be influenced by the gross motor functions.

Table 3 shows the description for the adult population. Each item consists of a five-level Likert scale, whose values are shown in Roman numerals, following the same representation. People included in the study had severe affectations in their mobility and neuromuscular functions, since the values shown in columns (chapter 7/b7) and (chapter 4/d4) are all equal to or higher than III. In particular, 65% and 85% of participants scored IV and V in chapter 4/d4 and chapter 7/b7, respectively.

**Figure 2:**
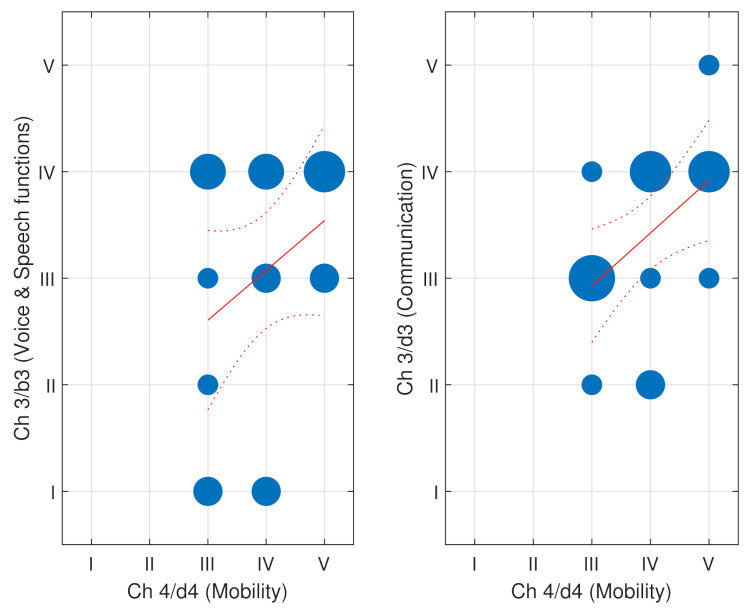
On the (**left**), bubble chart comparing the ICF items Ch3/b3 (voice and speech functions) to Ch4/d4 (mobility) of the assessed population in ASPACE. On the (**right**), the bubble chart comparing Ch3/d3 (communication) to Ch4/d4 (mobility). The sizes of the blue circles are proportional to the number of occurrences. The linear regression (solid line) and the confidence intervals (dotted line) are also shown in red.

Similarly, the adults in this set showed high impairments in their communication skills. In total, 50% of them were level IV or higher in both chapter 3/d3 and chapter 3/b3 items. Therefore, difficulties in voice and speech production consequently reduced the communicative capabilities of participants. Figure 2 depicts the bubble charts of these two parameters along with mobility (chapter 4 /d4). A relationship between communication (ch3/d3) and mobility (ch 4/d4) (*p*-value < 0.05) was found, but not between voice and speech functions (ch3/b3) and mobility function (*p*-value = 0.16). The level of voice and speech function was related directly and strongly to independence in communication (ch3/d3) (p<0.01). Furthermore, the limitations in mobility function (ch7/b7) were directly related to the independence in mobility activities (ch4/d4) (p<0.01).

We did not find any relationship between variables such as the gender or age of participants and level of mobility or communication skills assessed with the ICF and GMFCS, MACS and CFCS.

### 2.4. Discussion

The severity of mobility limitations that we found in both the young and adult sets was striking. Users with level I were not found in either group, and there were even no adult participants in level II. We needed to put these results in context with other published studies. In [19], a 4–11 year-old population in southern Sweden was analyzed. The prevalence in CP was 2.7 cases per 1000 inhabitants, and 48% of the CP population were level I. In [20], nine CP registries worldwide were shown, and the average proportions of each GMFCS level were 34.2%, 25.6%, 11.5%, 13.7% and 15.6% for levels I–V, respectively. Here, again, the proportions of levels I and II were higher than found in our study. In [6], participants were classified using the GMFCS, and the percentages of the population in each of the five levels were 27.8%, 12.2%, 18%, 20.5% and 20.8% for levels I–V, respectively. In [21], data were available on the motor abilities of the total population of children with CP in Scotland. The GMFCS level distribution was as follows: I—37.2%, II—18.2%, III—10.8%, IV—12.6% and V—21.2%. In comparison with those studies, our population showed a clear bias toward higher levels in the mobility scales, as the centers we cooperated with usually deal with seriously affected people, and the aforementioned studies included a much larger population. Regarding communication abilities, we found that 25% and 50% of the young and the adult groups showed very poor communication capabilities. We did not find a relationship between the CFCS and GMFCS in the young population. In other words, communication for this group is not influenced by motor skills. Important differences between the two groups may be due to the sample size and the fact that the two centers have different characteristics. For example, CEEEMS is a public center that also includes an official educational curriculum and a professional training program. Therefore, the people who attend both centers might have a slightly different profile.

## 3. Study 2: Regular Physical Activity in People with CP

Physical activity (PA) is defined “*as any bodily movement produced by skeletal muscles that results in energy expenditure*” [22]. Exercise or regular physical activity (RPA) is a type of PA that is planned, structured and repetitive and has the objective of improving people’s health. Increasing RPA in individuals with CP in childhood and continuing through adolescence is key to improving morbidity and mortality rates within this population. RPA produces benefits in motor functions in children [23,24,25] and adolescents with CP [26,27] and improves gait speed [28,29], spasticity [30], balance [31] and muscle strength [27,29].

Children with CP are 30% less engaged in PA than their normally-developed counterparts and two times more likely to be engaged in SB [32]. More than 75% of children and adults with CP have also been found to spend nearly all of their waking hours engaged in SBs [12]. Promoting adherence to RPA programs and a lifestyle incorporating exercise from an early age is crucial to avoid a progressive decrease in motor abilities during development [7] and aerobic fitness in adulthood [33].

The guidelines of the American College of Sports Medicine (ACSM) [34] recommend a frequency of 5 days/week of moderate exercise or 3 days/week of vigorous exercise for cardiorespiratory fitness and at least 2 days per week of muscle strengthening. For people with typical development who are not conditioned, the recommendation is to include light to moderate-intensity exercise and moderate and vigorous intensities. The recommendation is 20 to 60 min of continuous and rhythmic moderate or vigorous exercises that involve major muscle groups. Although the recommendation does not mention people with CP, nothing suggests a priori that these requirements should be different for them, and everyone needs physical fitness. These recommendations may be quite difficult to achieve and some exercises may be impossible to accomplish depending on the level of mobility. The positive aspect is that people with CP will reap health benefits even if they do less than the recommendations.

The authors in [12] conducted a thorough review of scientific literature based on studies in which participants with CP received cardiorespiratory endurance training and muscle strengthening versus a placebo or no intervention. Four parameters were investigated related to PA: the frequency, intensity, time and type of exercise.

- Frequency refers to the number of sessions per week. The ACSM recommendation includes at least three to five sessions to increase or maintain cardiorespiratory fitness with a recovery between sessions of 24–36 h. Some studies demonstrated that training was still effective although the frequency did not meet the minimal recommendations [35,36,37];
- Intensity is related to effort and is often indicated relative to maximal heart rate, heart rate reserve (HRR), the difference between a person’s measured or predicted maximum heart rate and resting heart rate and/or peak oxygen consumption. Although the intensity may vary from the beginning of the training, in general, all studies were aligned with the recommendation of more than 60% of maximum heart rate or more than 40% of the HRR;
- Time refers to the duration of the training session, which may be at least 20 min for aerobic workout and for a minimum of 8 or 16 consecutive weeks, depending on frequency;
- Types of activities are diverse and suited to people’s conditions (sports, running, muscle strengthening, etc.), and they must fulfill the therapist’s recommendations.

No specific duration for resistance training has been identified for effectiveness. The training period should last at least 12–16 consecutive weeks. Extra benefits in motor functions can be achieved by adding weight to exercise [38], which has also been proven efficient in improving bone health [39] and mineral density [40] in children with CP. Similarly, good results were also obtained when applying complementary techniques, such as functional electro-stimulation (FES), during strengthening exercises [41] and cycling [42], or a technique that makes the whole body vibrate when performing stretching exercise [43].

In this study, we attempted to gauge the current situation regarding PA for people with CP in ASPACE and CEEEMS. The main goal was to analyze the degree of fulfillment of the ACSM recommendations, the type of exercise they usually performed and the facilities and equipment required.

### 3.1. Participants

Forty people (21 female, 19 male) aged between 6 and 58 agreed to participate in this brief study (the same population as that in the previous study; for a more comprehensive characterization, see Table 2 and Table 3).

### 3.2. Methods

In this study, participants had to complete a questionnaire (shown in Table 4) with the support of their caregivers or familiars. The questionnaire contained items regarding the practice of PA, which included the frequency, intensity, duration, place, type of activity, etc.

#### Data Analysis

Non-parametric tests were applied in order to analyze the possible relation between variables: Spearman correlation for the quantitative variables, the U Mann-Whitney or Kruskal-Wallis tests to determine the differences in means, and the chi-square test for the qualitative variables. All hypothesis tests were set at p<0.05.

### 3.3. Results

Most of the enlisted people (38) performed RPA in the ASPACE or CEEEMS facilities, mainly for rehabilitation purposes, with a frequency of at least once a week. Only two participants did not perform any kind of regular activity due to the high level of anxiety that PA induced in them.

**Figure 3:**
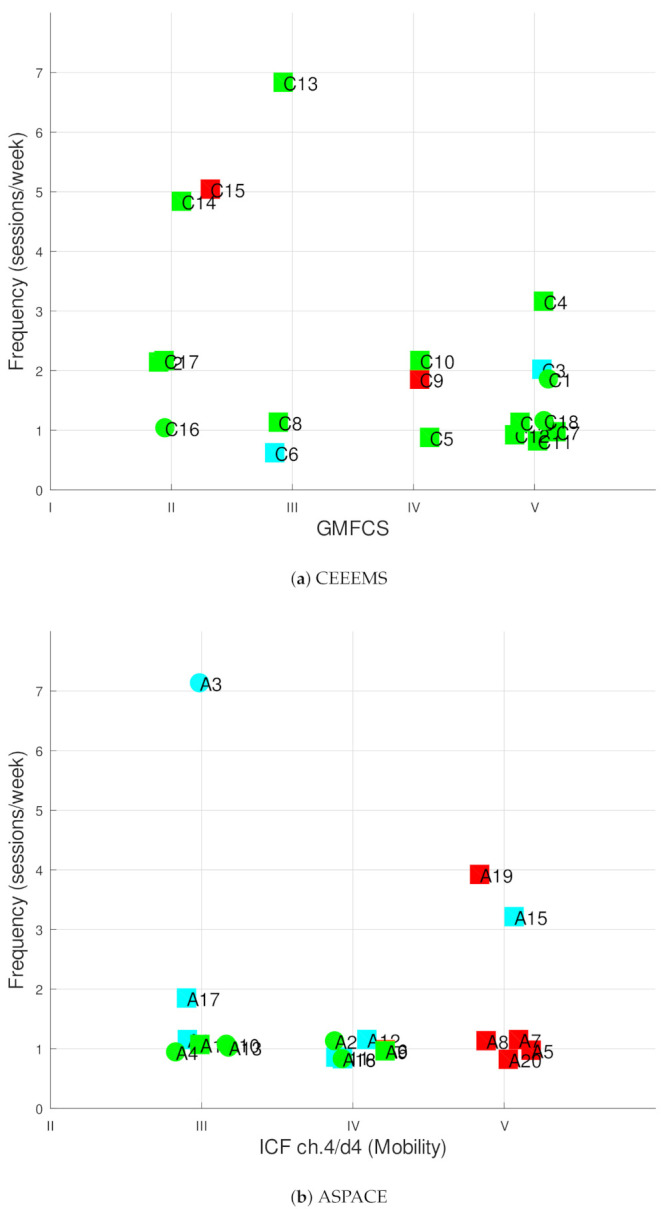
Frequency of RPA sorted by disability level for all participants in (**a**) CEEEMS and (**b**) ASPACE. The shape of the marker represents the type of activity: rehabilitation (squares) or rehabilitation + personal choice (circles). The color also indicates the degree of acceptance of the activity: like it (green), neutral (blue), not like it (red).

In ASPACE, participants A17, A15, A19 and A3 did additional exercise at home (three participants) or at specialist centers (two participants) with varying frequency: once, twice or three times a week or every day, respectively. In general, sessions were of light intensity, with a duration of 45 min, and only subject A3, who also attends a hydrotherapy session once a week, performed moderate exercise on a habitual basis. In CEEEMS, most of the participants did moderate exercise (79%) and only one of them, C13, performed additional exercise at home. Figure 3 depicts the frequency, type of activity and degree of acceptance of particpants, sorted by motor skills and centers, while Table 5 shows the survey results.

Regarding motivation, 55% of respondents liked performing PA or liked it very much. In contrast, 22.5% of the polled population did not like it, and the remaining 22.5% selected the neutral option. According to the ICF chapter 4/d4 level, the ASPACE sample test found that that the higher the ICF, the higher the percentage of dislikes. In particular, proportions of 0%, 14.3% and 83.3% were found for levels III, IV and V, respectively, which can also be seen in Figure 3b, where most of the red shapes are located on the right. In CEEEMS, people like doing sport (percentages of 66% for levels III and IV) and slightly higher at levels II and V ( 80% and 87.5%).

Nine respondents included personal choice, together with the rehabilitation of motor functions, as the reason for their RPA. In this subgroup, no negative motivation was found, but surprisingly, only one woman and one girl performed RPA apart from their daily rehabilitation program. In ASPACE, the personal choice option was found to diminish as motor functions decreased. In particular, 57.1%, 28.6% and 0% of respondents for levels III, IV and V, respectively, reported that doing exercise was a personal choice. Conversely, in CEEEMS, higher ratios were found at GMFCS levels V and II (25% and 20% of respondents) which agreed with the higher ratios obtained in motivation at the same levels. For the remaining levels at CEEEMS, no personal choice option was found among the answers.

We also analyzed how motor and voice and speech skills (assessed with the ICF and the GMFCS, MACS and CFCS) or the independence in mobility and communication influence the characteristics of PA, including frequency, intensity and type of activity and motivation. We did not find any significant relationship.

Taking into account the differences concerning the age and daily activities of the two groups (the mean age was 38 in ASPACE and 16.05 in CEEEMS), we considered it important to analyze the data separately to gain a specific perspective on PA and its influence on mobility and communication levels in persons with CP in different vital stages.

In the case of ASPACE, the motivation for performing and participating in PA was only related significantly to the type of activity (p<0.05). Therefore, participants who only performed PA for rehabilitation had low motivation, while people who carried out PA for rehabilitation and their own choice (leisure) showed higher levels of motivation.

The relation between motivation to perform PA and the type of activity (and intention) was more prevalent in the case of participants from CEEEMS (p<0.01). Moreover, the motivation in this group was related to the intensity of the PA (p<0.01). People that performed moderate levels of PA seemed to have higher motivation than those that did low intensity PA.

The frequency of the PA did not seem to have any influence on the motivation for doing the activity.

A final part of this study was to identify the equipment that people with CP usually need to do several activities. We found the following to be required: static powered/non-powered bikes for upper and lower-limb exercises, standers and walkers for gait exercises and a spirometer for respiratory therapy. We did not find any software application for promoting PA apart from participants’ own caloric-meters and pedometers included in some equipment to monitor their amount of exercise.

### 3.4. Conclusions

The survey revealed that most people with CP did not fulfill the recommendations of the ACSM for RPA in terms of frequency and accrued time. The age, gender or characteristics of mobility and communication (determined with the ICF and GMFCS, MACS and CFCS) were not related with this activity.

We found that motivation was the only variable that had any influence on participation in PA. Namely, in ASPACE, people who attended only rehabilitation sessions admitted not liking doing exercise. In CEEEMS, motivation was found to influence the type of activity and the intensity of the exercise.

## 4. Study 3: Review of Physical Activities

In this section, we conducted a review of PA to find the type of exercises and gadgets or specific equipment that had proven their worth for people with CP, along with the devices necessary for performing and monitoring PA, with a focus on low-cost solutions.

### 4.1. Methods

We conducted a procedure similar to a *“scoping review”* to find the kind of PA performed most often [44] by participants with CP. Data were obtained from the Scopus database (https://www.scopus.com (accessed on 20 August 2021)), using the keywords “cerebral palsy” AND “physical activity” and limiting the results to the years from 2019 to 2021. The search returned 168 items. In the first phase, we screened exclusively the title and abstract; subsequently, the remaining articles went through a full-text reading process. As a result, 45 papers were selected for this study. From these, we extracted, on the one hand, a set of efficient exercises suited to every level of motor functionality and, on the other hand, the type of equipment used for monitoring PA.

### 4.2. Results

#### 4.2.1. Recommended Exercises and Equipment

As explained above, the negative effects of SB can be mitigated by promoting different PAs. Some of the exercises required specific equipment, although not always. Indeed, large numbers of exercises can be found in the scientific literature that have demonstrated their efficiency in improving cardio-respiratory and muscular endurance without the need for any special equipment. For instance, for people with CP in GMFCS level I, or the equivalent in the ICF scale, these exercises include running [45], the shuttle-run game [46] or dancing [47]; for GMFCS I–III, activities included walking (gait) [45], stepping up-down [26,48], sit-to-stand, crawling [42] and squatting [49]; for levels up to GMFCS level IV, we added the strengthening interventions of the knee extensor, hip flexor and ankle dorsiflexors [43].

Other studies included some equipment that is useful for training and promoting PA in people with CP: an elliptical machine for walking [48] or a treadmill [26] for running, both of which are appropriate for participants with GMFCS levels I–III. For levels I–IV, other authors employed adapted bikes for cycling [42,50,51] or a feet ergometer [27]. Other efficient exercises can be done on a physiotherpy ball (bouncing, high sitting, reaching a toy, etc.) [52]; in a universal exercise unit (UEU) [53,54], a specialized piece of equipment allows the therapist to isolate specific muscles and improve a child’s strength (GMFCS levels II–IV); and elastic bands can be used for training upper limbs [55,56] for people with CP in levels I-III. Table 6 contains a broad catalog of activities that have demonstrated their validity.

Playing sports is an enjoyable, efficient and involving alternative for improving participants’ abilities [57]. Several sports have been analyzed for children with CP [57,58]. In particular, for level I in the GMFCS, skating, martial arts and table tennis are recommended, up to level II, football is recommended; up to level III, swimming is recommended; and for levels III–IV, gymnastics and hockey with electric wheelchairs are recommended.

The use of active video-games (which require interaction through a hand/foot controller) may be more enjoyable for users when exercising and may represent a good complement to traditional face-to-face rehabilitation interventions [59], and they can be done at home. In general, commercial video-games are not suitable for people with disabilities. However, some solutions have been developed to reduce this gap. In particular, the Xbox Adaptive Controller provides players with a customized controller, or Kinect [60,61] has demonstrated efficacy in improving motor functions in individuals with levels I–III in GMFCS. Other games can easily be played by this population [62] or designed considering the universal access principle, which means that the game can be used by any user [63]. For example, the Nintendo’s Wii controller does not need any kind of adaption and improves grip strength [64]. The Nintendo Wii-Fit Balance Board has also been used to enhance dynamic balance in people with CP with GMFCS levels I–II [65,66]. Virtual reality platforms, such as PlayStationVR and SteamVR, have a wide catalog of video-games which could also be used to promote PA with a positive effect on motor functions [59,67,68]. Hence, video-games are flexible tools that can be adapted easily to meet user skills [59,69].

physical activity (PA) through therapies involving animals can be another interesting option that is more entertaining and that has significant benefits on body control [70,71,72].

#### 4.2.2. Equipment for Monitoring Physical Activity

Determining the performance of a PA is an important topic in the assessment of the efficiency of therapy. The inertial measurement unit (IMU) (accelerometers and gyroscopes) is one of the most widely used devices for monitoring interventions, because it weighs little and is cheap, small, unobtrusive and easy to fit [57,69,73,74]. IMUs measure the acceleration associated to a person’s movement wherever they are placed, allowing an estimation of the action performed and the subject’s posture [50,75]. The information delivered by these devices is directly linked to intensity of the PA. Accelerometers can therefore be used to estimate energy expenditure during PA [76]. The placement of these devices depends on the type of activity to be measured and the subject’s abilities [50,77,78]. When individuals have problems standing up, the accelerometer is normally placed on the chest or on the wrist of the dominant hand [42,77]. As mentioned briefly above, these devices can be used to distinguish between sedentary periods, intensity levels in PA and different kinds of activities. In [50], a commercial accelerometer was analyzed to monitor and classify up to six different kind of PA (non-wear, sitting, standing, walking, cycling or running), concluding that the use of this sensor was feasible. A similar study, but in this case based on using machine learning and two accelerometers, showed that five different activities (rest, upper-limb task, walking, wheelchair displacement and cycling) could be distinguished to evaluate the efficiency of an activity [79].

Electromyography (EMG) and acoustic myography allow the recording of muscle activity during PA. With this equipment, different parameters can be evaluated: spatial and temporal fiber recruitment, contraction intensity, fatigue, etc. These devices revealed that participants with CP needed more muscle fibers to be activated to maintain exercise intensity compared to healthy individuals [80,81,82].

Another important physiological signal used to evaluate intensity and aerobic performance in PA and discriminate resting from task periods was the heart rate (HR). A video-game could use this information to reduce the intensity of an activity [83], adapting the PA to the users’ capabilities and preventing their health from being put at risk [75,84].

Another alternative is the use of wearable smart devices (bands, watches and phones) [85]. These devices have several measurement tools—a pedometer, heart rate monitor, sleep monitor, etc.—and have demonstrated their effectiveness for analyzing gait, balance and range of motion (ROM), although their accuracy needs to be improved [86].

### 4.3. Discussion of Low-Cost Solutions

Setting up a PA routine is one the first actions required to prevent participants’ health from worsening, and here, financial considerations also come into play. Table 6 contains several PAs that have exhibited effectiveness in promoting active lifestyles in people with CP. However, the cost of activities may be a handicap for RPA: for example, walking on a treadmill, cycling using adapted bikes and electric wheelchair hockey involve high outlays. Thus, low-cost solutions need to be provided whenever possible. Activities such as crawling, walking, running, playing shuttle-run games, doing sit-to-stand tasks, stepping up-down, squatting, etc. can boost an active routine of physical exercise and do not need any special equipment (they are free). Some exercises use inexpensive tools that can be used at home: an elastic band, physiotherapy ball, feet ergometer, etc. Other physical activities require several elements that involve more expense; e.g., swimming costumes and facilities are necessary to go swimming. When using a computer, if the cost of the computer itself is not considered, hardware and/or software can be purchased at a relatively low cost. For example, some online exercise services can be accessed by subscription, with a monthly fixed cost. The Kinect device has been used in the scientific literature to monitor movements through the location of people’s joints in a 3D environment. However, Kinect was discontinued. Webcams provide a cheap and readily available alternative, and a software application called OpenPose [87] locates individuals’ joints in a similar way to Kinect. The Nintendo Wii controller has also demonstrated potential for PA, and it is possible to acquire inexpensive compatible devices.

The monitoring of PA is necessary to assess participants’ progress and adapt their exercise accordingly, which can be done via subjective analysis (therapist and/or data from family) or by more objective and affordable commercial gadgets such as smartphones, smartwatches or activity monitoring devices. As activities are registered, potential health risks must be limited. Smartwatches for sports normally include heart rate (HR) measurement which gives valuable information about exercise intensity. A cheaper alternative to these devices is to assemble them. For example, using Arduino, it is possible to develop an activity monitoring device based on an IMU or implement a shield to register electrocardiography (ECG) [88].

## 5. Global Discussion

Participants in this study had more severe impairments affecting their mobility than the users included in other studies in the literature [6,19,20,21], with a population in level I ranging from 27.1% up to 48%. In our study, we did not find any person at that level, possibly because the centers we cooperated with usually deal with seriously affected people, while the aforementioned studies included a much larger population. The percentage of people rated with levels IV and V ranged from 29.3% to 41.3%, which was in line with the 35.3% in CEEEMS and very far from the 65% in ASPACE. Regarding communication abilities, we found a great variation among our participants. In the case of young people, 75% of them had sufficient communication capabilities (Levels I, II, III), while for adults, the percentage at the same level dropped to 50%.

According to our findings, most of the respondents in Study 2 did not fulfill the minimum ACSM recommendations for RPA. Only A3, A15 and A19 in ASPACE and C4, C13, C14 and C15 in CEEEMS (17.5% of the total) accumulated more than 150 min of exercise a week in three different sessions. Benefits for health and motor functions have been reported with more relaxed conditions. In [12], the authors performed a systematic review analyzing the effects of exercise interventions; as a conclusion, they suggested a minimum of 20 min in at least 3 sessions/week at 40% of HRR or 60% of maximum HR. If we follow these slightly more relaxed guidelines, six participants could be added to the group above, although with a lower number of sessions per week. Therefore, 67.5% of participants who only attended one 45 min or 30 min session a week did not fulfill the reduced recommendation either.

Participants’ skills affect PA quality and movements [74]. The level of GMFCS may affect the intensity and number of repetitions of exercises [89]. Thus, the lower the level in the GMFCS, the higher the intensity, number of repetitions, complexity and variety of activities that can be done. However, the mobility level is not always linked to how active a person is. Some people with low mobility show higher levels of PA than other people with better motor functions and with a SB [77]. Most people at ASPACE did light-intensity exercise and required a complete therapist support (45%) or frequent help (25%), which is explained by the severity of their mobility impairments. Only one participant, who was assessed as level III, usually performed moderate exercise (5%). This finding was in line with previous studies such as [90,91], in which only a low percentage of people with CP were engaged in moderate to vigorous activity (only present in GMFCS levels I–III).

In Study 3, we listed a broad catalog of activities that have proven their worth for benefiting health and fitness: walking (freely or on an elliptical or treadmill machine), cycling, using a feet ergometer or elastic bands, playing sports such as swimming or hockey, playing video-games or doing activities with animals. Most of these were not feasible for people with severe motor disabilities, such as, for example, those with level IV and V in GMFCS, who spend a great deal of time sitting [92]. Some findings demonstrated that frequently interrupting or replacing SB with some light-intensity exercise [93,94,95] or brief and frequent daily PA [33] has enormous benefits on health.

Promoting PA should be a priority to prevent a worsening of participants’ health. RPA, whatever its intensity, has a positive effect on balance, muscle strength and gait speed [27,28,29] and has benefits proportional to the time dedication and frequency [96]. Apart from that, a person’s motivation to engage in PA is essential for them to be fully involved in the activity. In this sense, our study showed that the participant’s motivation conditions the frequency, intensity and type of PA activities.

Health professionals, people with CP and relatives should work together to create a lifestyle and promote adherence to PA programs [97], which should include an appropriate exercise routine, avoid fatigue and prevent adverse events [80,98,99,100]. In general, exercise is seen as central to improving mobility and fitness, and facilities, programs and attitudes towards it are key features for success [101]. Extending the time spent doing PA outside the centers could also be positive. Exercising at home will most likely need family support [102] and an appropriate program which must be safe and not require the supervision of a health professional. Structured, home-based exercise programs have become more efficient at improving gait [103], general mobility and the realization of daily activities [104] regardless of whether the program was given on paper or online [105].

Interventions including the participation in competitive or collaborative groups, the use of music during exercise or dance-based therapies have also demonstrated their efficacy. For example, playing boccia or hockey in powered wheelchairs [57] has led to improvements in the performance of daily activities in adults [40], motor functions of children [106] and the quality of life of children and young adults and health conditions in general [38]. Significant increases in muscle strength and walking speed were found in [107] when rousing music was played during a resistance exercise. Improvements in the ROM of the hip and ankle, stride length, walking speed, cadence and step can also be found in interventions in which participants completed a dance-based exercise [47].

Another feasible solution for promoting and increasing the frequency in RPA was the use of virtual assistants. These applications have several sensors that monitor the quality and duration of the exercise, allowing the activity to be performed at home and to be adapted to maintain certain physiological variables in an appropriate range [108]. A system such as virtual exercise rehabilitation assistant (VERA) has been shown to be very efficient in detecting and reporting information about RPA. VERA uses a laptop and a Kinect to detect movements [109]. The use of robots to promote PA for the elderly has also been investigated [110], and they were found to be more motivating than virtual agents [111,112].

### Study Limitations

The main limitation of this research was the size of the sample and the fact that it was divided into two sub-groups. Thus, the data obtained have to be treated with caution, taking into account the specific Spanish contexts and the special characteristics of the centers from which the sample population came. Apart from that, the main difference between the two sub-groups was the mean age (22 years old), so to analyze certain variables, such as the motivation, intensity or frequency of PA, the research group had to perform the test separately in order to correctly identify the presence of significant relationships.

The use of two different measures to assess the level of mobility and communication skills of participants (the ICF for ASPACE, and the GMFCS, MACS and CFCS for CEEEMS) could be another limiting factor, due to the difficulty of analyzing the data jointly. That fact was conditioned by the specific assessment tools used independently in each collaborating center. For future research, the assessment and classification of the population should be homogenized to obtain stronger results.

Finally, the third study was based on identifying, through a scoping review, the best and most widely-used exercises and equipment for PA for people with CP. Therefore, the results do not reflect the actual use or consequences on PA for participants in Studies 1 and 2. To give a complete overview of the implications for our sample, future research has to contemplate this goal and be oriented towards analyzing the results obtained after the application of a specific sequence of exercises and/or equipment for PA.

## 6. Conclusions and Future Work

People recruited in this work had a higher average level of motor dysfunction than in other published studies. We hypothesized that the main reason for this difference is that the centers assist people who have a greater need for specialized attention.

We found that most of the participants did not meet either the ACSM guidelines for RPA or the more relaxed recommendations that have proven their efficacy in previous studies. The frequency, intensity and accrued weekly time were mostly unfulfilled.

Our findings revealed that adult people with poor motor functions dislike doing exercise, and the underlying reasons for this must be faced in future research. Exercising in groups with music, dancing, playing games, performing activities at home, playing with balls and using elastic bands are feasible and costless options that can improve QoL and fitness.

This research needs to be extended in the near future to a greater number of people, applying interventions that break SB, increase the practice of PA and introduce elements that assess the quality of the exercise (wearables, smart phones, accelerometers, Kinect, etc).

## Figures and Tables

**Table 1:** Motor functions and communicative skills included in the ICF scale.

Body Functions		
**ID**	**Name**	**Definition**
Ch 3/b3	Voice and speech functions	Functions of producing sounds and speech.
Ch 7/b7	Neuromusculoskeletal and movement-related functions	Functions of movement and mobility, including functions of joints, bones, reflexes and muscles.
**Activities and participation**		
**ID**	**Name**	**Definition**
Ch 3/d3	Communication	General and specific features of communicating by language, signs and symbols, including receiving and producing messages, carrying on conversations, and using communication devices and techniques.
Ch 4/d4	Mobility	Moving by changing body position or location or by transferring from one place to another, by carrying, moving or manipulating objects, by walking, running or climbing, and by using various forms of transportation.

**Table 2:** Children’s Assessment in Centro Específico de Educación Especial Mercedes Sanromá (CEEEMS), including gender and age.

Subject	Gender	Age	CFCS	MACS	GMFCS
C01	F	18	III	IV	V
C02	M	6	III	III	II
C03	M	15	III	IV	V
C04	M	8	III	IV	V
C05	M	18	I	I	IV
C06	F	18	I	II	III
C07	F	19	III	V	V
C08	F	19	I	III	III
C09	M	18	II	III	IV
C10	M	12	IV	IV	IV
C11	M	14	III	V	V
C12	M	17	IV	IV	V
C13	F	18	I	II	III
C14	F	19	II	II	II
C15	F	19	III	III	II
C16	M	10	IV	III	II
C17	M	20	V	III	II
C18	F	18	IV	V	V
C19	F	21	III	IV	V
C20	M	14	I	III	III

**Table 3:** Adult Assessment according to ICF scale used in ASPACE, including gender and age. A 5- level Lickert scale, whose values have been shown in Roman numerals, following the same representation than in Table 2, is used to assess the items grade.

			ICF			
			MAC and GMFCS		CFCS	
**Subject**	**Gender**	**Age**	**Ch. 7/b7** **(Neuromusculoskeletal** **and Movement-Related** **Functions)**	**Ch. 4/d4** **(Mobitliy)**	**Ch. 3/b3** **(Voice and Speech** **Functions)**	**Ch. 3/d3** **(Communication)**
A01	F	51	III	III	IV	III
A02	F	27	IV	IV	IV	IV
A03	M	32	IV	III	II	III
A04	F	36	IV	III	I	II
A05	F	33	IV	V	IV	IV
A06	M	50	V	IV	III	IV
A07	M	58	IV	V	III	III
A08	M	24	V	V	IV	IV
A09	F	49	IV	IV	I	II
A10	M	35	III	III	IV	III
A11	F	35	IV	IV	III	III
A12	M	42	IV	IV	I	II
A13	M	46	III	III	III	III
A14	M	43	IV	III	IV	IV
A15	F	51	IV	V	IV	V
A16	F	28	IV	IV	IV	IV
A17	F	26	IV	III	I	III
A18	F	33	IV	IV	IV	IV
A19	M	26	V	V	IV	IV
A20	F	35	IV	V	III	IV

**Table 4:** Survey given to people with CP, relatives and health professionals.

ID	Question
1	Sex.
2	Age.
3	Family environment.
4	Do you do physical activity? (Yes/No). *If so, please continue with the survey, otherwise, you have just finished.*
5	Indicate how often do you workout (number of days and sessions a week).
6	How long does the session usually take? (min)
7	Select its intensity (Light, Moderate, Vigorous).
8	Where do you usually do exercise? (At home, at a specific center, outdoors, others)
9	What is the purpose of your physical activity? (Rehabilitation, personal choice, both)
10	Score your motivation to do physical activity. (I do not like it at all, I do not like it, Neutral, I like it, I like it very much)
11	Do you use any kind of supporting device or gadget to do the activity? If so, please describe it.
12	Do you use any kind of software to do exercise? If so, please describe it.
13	Please give any further information you consider relevant for this survey.

**Table 5:** Characteristics of PA routine followed by participants according to the survey questions (5, 7, 9 and 10).

		Gender		
		**Sample**	**Male**	**Female**
**Survey Questions**	**Possible Answers**	**N**	**N**	**N**
	Never	2	2	0
	Once per week	24	11	13
Frequency of PA	Twice per week	7	4	3
(5)	Three times per week	2	1	1
	More than three times per week	5	2	3
Intensity	Light	22	10	12
(7)	Moderate	16	8	8
Type of activity	Rehabilitation	29	15	14
(9)	RHB and personal choice	9	3	6
	I like it very much	4	2	2
	I like it	19	10	9
Motivation	Neutral	9	4	5
(10)	I do not like	5	3	2
	I do not like at all	3	1	2

**Table 6:** Overview of physical activities by motor functionality. Each level contains the activities of higher level.

Level			Activities		
I					Crawling, running, shuttle-run game, dancing, martial arts, skating, playing table tennis.
	II				Football, videogames (Nintendo’s Wii controller and Wii-Fit Balance Board).
		III			Sit-to-stand, step up-down, squatting, walking (free, gait, elliptical, treadmill), elastic bands for training upper limbs, swimming, playing videogames (Microsoft Kinect).
			IV		Strengthening of knee extensor, hip flexor, and ankle dorsiflexors, physio-ball exercises, universal exercise unit, feet ergometer, cycling using adapted bikes, wheelchair hockey, gymnastics, animal therapy.
				V	Virtual-reality games.
